# Prognostic model of long-term advanced stage (IIIB-IV) EGFR mutated non-small cell lung cancer (NSCLC) survivors using real-life data

**DOI:** 10.1186/s12885-021-08713-8

**Published:** 2021-08-31

**Authors:** Lourdes Gutiérrez, Ana Royuela, Enric Carcereny, Rafael López-Castro, Delvys Rodríguez-Abreu, Bartomeu Massuti, José Luis González-Larriba, Rosario García-Campelo, Joaquim Bosch-Barrera, María Guirado, Carlos Camps, Manuel Dómine, Reyes Bernabé, Joaquín Casal, Juana Oramas, Ana Laura Ortega, Mª. Angeles Sala, Airam Padilla, David Aguiar, Oscar Juan-Vidal, Remei Blanco, Edel del Barco, Natividad Martínez-Banaclocha, Gretel Benítez, Blanca de Vega, Ainhoa Hernández, Maria Saigi, Fernando Franco, Mariano Provencio

**Affiliations:** 1 Medical Oncology Department, Hospital Universitario Puerta de Hierro, Calle Joaquín Rodrigo n1, 28222, Majadahonda, Madrid, Spain; 2 Biostatistics Unit, Puerta de Hierro Biomedical Research Institute (IDIPHISA), CIBERESP, Madrid, Spain; 3 Catalan Institute of Oncology, Hospital Universitari Germans Trias i Pujol, B-ARGO, IGTP, 08916 Badalona, Barcelona Spain; 4 Hospital Clínico Universitario de Valladolid, 47003 Valladolid, Spain; 5 Hospital Universitario Insular de Gran Canaria, 35016 Las Palmas de Gran Canaria, Spain; 6 Hospital General Universitario de Alicante, 03010 Alicante, Spain; 7 Hospital Clínico San Carlos, 28040 Madrid, Spain; 8 Complexo Hospitalario Universitario A Coruña, 15006 A Coruña, Spain; 9 Catalan Institute of Oncology, Hospital Universitari Dr. Josep Trueta 17007, Girona, Spain; 10 Hospital General Universitario de Elche, 03203 Elche, Alicante Spain; 11 Hospital General Universitario de Valencia, Universitat De València, CIBERONC, 46014 Valencia, Spain; 12 Hospital Universitario Fundación Jiménez Diaz, IIS-FJD, 28040 Madrid, Spain; 13 Hospital Universitario Virgen del Rocío, 41013 Sevilla, Spain; 14 Hospital Universitario de Canarias, 38320 San Cristóbal de La Laguna, Santa Cruz de Tenerife, Spain; 15 Hospital Universitario de Jaén, 23007 Jaén, Spain; 16 Hospital Universitario Basurto - OSI Bilbao Basurto, 48013 Bilbao, Spain; 17 Hospital Universitario Nuestra Señora de la Candelaria, 38010 Santa Cruz de Tenerife, Spain; 18 Hospital Universitario de Gran Canaria Dr. Negrín, 35010 Las Palmas de Gran Canaria, Las Palmas, Spain; 19 Hospital Universitario y Politécnico La Fe, 46026 Valencia, Spain; 20 Oncology Service, Consorci Sanitari de Terrassa, 08191 Rubí, Barcelona, Spain; 21 Hospital Clínico Universitario de Salamanca, 37007 Salamanca, Spain; 22 Hospital Universitario Puerta de Hierro, 28222 Majadahonda, Madrid, Spain

**Keywords:** Non-small cell lung cancer, EGFR, Predictive modeling, Nomogram, Long survival

## Abstract

**Background:**

There is a lack of useful diagnostic tools to identify EGFR mutated NSCLC patients with long-term survival. This study develops a prognostic model using real world data to assist clinicians to predict survival beyond 24 months.

**Methods:**

EGFR mutated stage IIIB and IV NSCLC patients diagnosed between January 2009 and December 2017 included in the Spanish Lung Cancer Group (SLCG) thoracic tumor registry. Long-term survival was defined as being alive 24 months after diagnosis. A multivariable prognostic model was carried out using binary logistic regression and internal validation through bootstrapping. A nomogram was developed to facilitate the interpretation and applicability of the model.

**Results:**

505 of the 961 EGFR mutated patients identified in the registry were included, with a median survival of 27.73 months. Factors associated with overall survival longer than 24 months were: being a woman (OR 1.78); absence of the exon 20 insertion mutation (OR 2.77); functional status (ECOG 0–1) (OR 4.92); absence of central nervous system metastases (OR 2.22), absence of liver metastases (OR 1.90) or adrenal involvement (OR 2.35) and low number of metastatic sites (OR 1.22). The model had a good internal validation with a calibration slope equal to 0.781 and discrimination (optimism corrected C-index 0.680).

**Conclusions:**

Survival greater than 24 months can be predicted from six pre-treatment clinicopathological variables. The model has a good discrimination ability. We hypothesized that this model could help the selection of the best treatment sequence in EGFR mutation NSCLC patients.

**Supplementary Information:**

The online version contains supplementary material available at 10.1186/s12885-021-08713-8.

## Background

Lung cancer continues to be the leading cause of cancer death, with 20–25% of deaths occurring in non-smoking patients, these usually being the cases with mutations in driver genes such as the Epidermal Growth Factor Receptor (EGFR). In the Asian population, the prevalence of the EGFR mutation is 40–50% however in US and Europe is about 15–20% [1]. In Spain, it is around 16% [2]. Overall survival of patients with EGFR mutations continues to improve due to the appearance of different generations of tyrosine kinase inhibitors (TKIs). Osimertinib in first line has achieved the best progression-free survival (PFS) and overall survival (OS) data in the FLAURA study [3].

There is no standard definition of a long survivor in lung cancer and different cut-off points are found in the literature [4, 5]. The median of survival in the first pivotal clinical trials with TKIs is over 24 months [6]. Randomized controlled trials (RCT) are considered the gold standard of evidence-based medicine. However, populations included may not be representative patients in real life considering that in many cases, these are older, have a poorer performance status (PS), rare mutations and with brain metastases detected more frequently at diagnosis [7]. For this reason, patient registry serves as real world data studies to verify the results obtained in RCT rather than carrying out phase IV studies in the clinical setting. These permit the inclusion of large numbers of patients, with longer follow-up than RCTs [8, 9].

In 2016, the Spanish Lung Cancer Group (SLCG) began a cooperative epidemiological lung cancer registry, with over 500 participating members from at least 78 hospitals in Spain. This registry is a large database containing over 15,000 cases of lung cancer.

This study aims to identifiy the characteristics present at diagnosis of these EGFR mutated patients that are associated with long survival.

## Materials and methods

This is an observational, multicenter; retrospective study that updates prospective follow-up data in the population of EGFR mutated lung cancer patients from Spanish hospitals participating in the SLCG thoracic tumor registry. The registry was approved by the Ethics Committee of Puerta de Hierro University Hospital (Majadahonda, Madrid) (No. PI 148/15) and is registered in the ClinicalTrials.gov database (NCT02941458). The study was carried out in accordance with the Helsinki Declaration.

All patients included had histological confirmation of lung cancer and the presence of an EGFR mutation. Cases were included systematically by hospitals participating in the registry. Data was collected via an online questionnaire with the following sections: a) demographic data; b) smoking history, categorized as never-smokers (< 100 lifetime cigarettes), former smokers or ex-smokers (quit > 1 year prior to diagnosis) or current smokers (continued smoking within 1 year of diagnosis) [10], occupation and family history; c) tumor characteristics at diagnosis, including the specific type of mutation and metastatic sites; d) treatments received, including detailed information on each (start and end dates); e) dates of tumor progression and sites; f) survival data.

After reviewing different publications (real-world data and pivotal studies of the main approved treatments) [11] in addition to the development timeline of our study (between 2009-2017), we consider that patients with advanced lung cancer and EGFR mutation, can be defined as long survivors when the overall survival (OS) is greater than 2 years (> 24 months).

### Study design and population

**Fig. 1:**
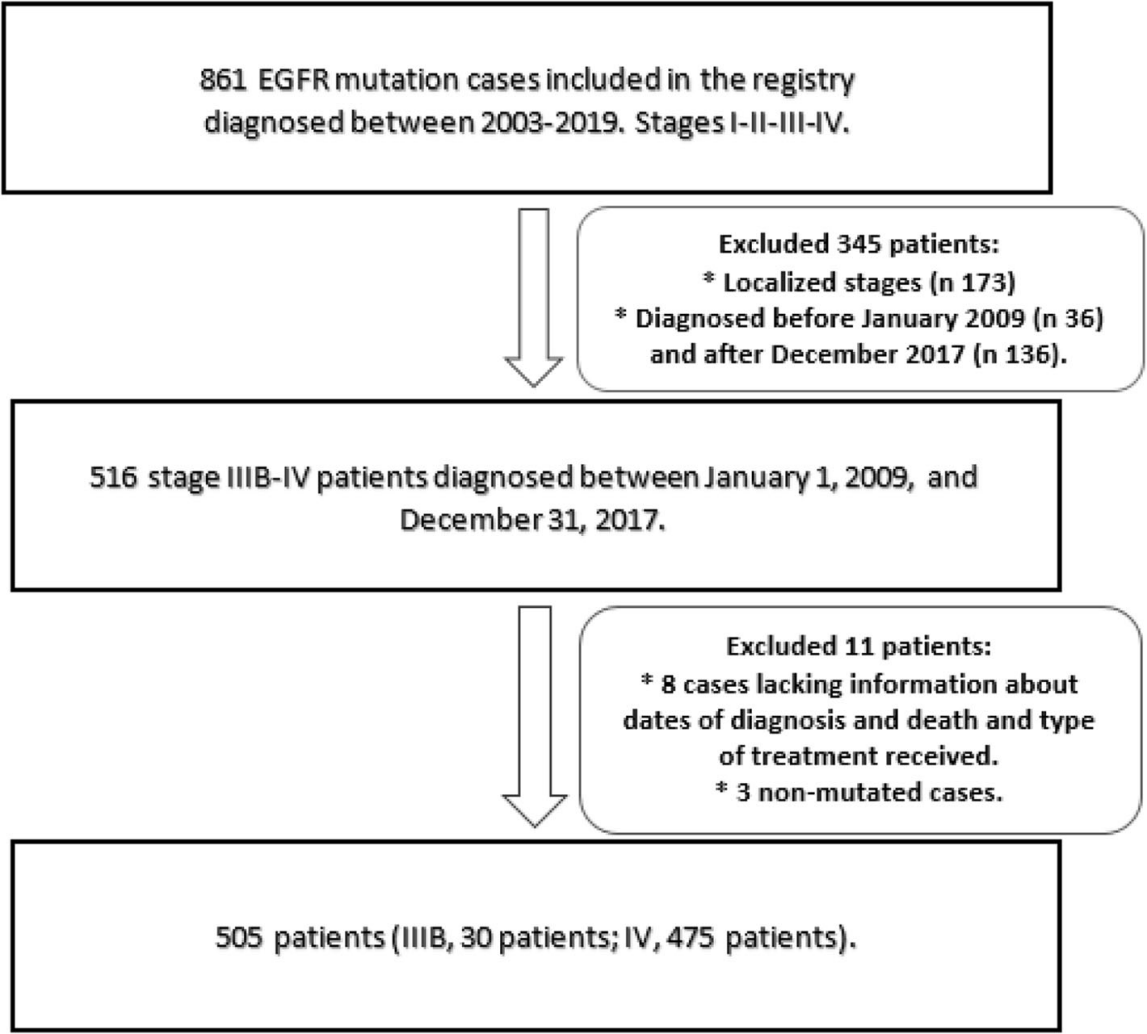
Flow chart of the study population selection

Firstly, all cases with EGFR mutation (any type of EGFR mutation and any stage) were collected from the SLCG thoracic tumor registry (Fig. 1). EGFR mutations were detected using the Cobas EGFR assay, a real time PCR test that identifies mutations in exons 18, 19, 20 and 21. In 20 cases, the specific mutation was not available. Only advanced stages IIIB-IV were included. Patients diagnosed before January 2009 were excluded to avoid older cases that had not had been treated with a TKI at any time during their evolution, as well as those diagnosed after December 2017 to guarantee at least 24 months of follow-up. Patients for whom the dates of last follow-up or death were not available were excluded as their survival could not be calculated. Most of the patients included with EGFR mutations were unable to receive osimertinib in first line as it had recently been approved. Therefore, the long-term survivors included, have received different treatment sequences with 1st and 2nd generation TKIs and chemotherapy.

### Statistical analysis

A descriptive analysis of the categorical variables was performed using absolute and relative frequencies and, for the numerical variables, using mean and standard deviation or median and percentiles 25 and 75, according to compliance with the normality assumption. OS was defined as time from diagnosis to death for any cause or end of follow-up (May 2020). Median follow-up was estimated using the reverse Kaplan-Meier method along with the 95% confidence interval [12]. The OS curve was estimated using the Kaplan-Meier method.

A long-term survivor was considered to be any patient still alive at 24 months from time of diagnosis. The univariate analysis to identify factors associated with survival at > 24 months was carried out using binary logistic regression.

Variables collected at diagnosis, before the start of treatment, were used for the multivariable analysis. A multivariable analysis was carried out using binary logistic regression for those variables that were significant in the univariate analysis, as well as others in the literature that have shown an association with survival.

A collinearity diagnosis and an automatic backward elimination (threshold to retain a variable *p* < 0.10) regression modeling strategy were performed, estimating the odds ratio (OR) and their corresponding 95% confidence intervals (95%CI). A nomogram was developed to improve the interpretation and application of the prognostic model in routine clinical practice[13].

Once the final model was obtained, its internal validity was evaluated via calibration and discrimination. Resampling techniques were performed by bootstrapping, with 500 replications. To measure the calibration, a calibration plot was generated in which the quintiles of the observed and expected event risks were graphically confronted. With perfect calibration, the line between the two risks would lie along the main diagonal of the plot. Discrimination was measured using the C-index, this being an analog of the area under the ROC Curve (AUC ROC), with values ​​ranging from 0.5 for no discrimination to 1.0 for perfect discrimination [14, 15].

The level of significance was set at 0.05, except for the exceptions previously described. The statistical package used was the Stata/IC v.16 (StataCorp. 2019. Stata Statistical Software: Release 16. College Station, TX: StataCorp LLC.).

## Results

### Study population characteristics

Of the 861 EGFR mutated cases found in the registry, 516 cases were selected. Of these, 8 were excluded due to the lack of data essential for calculating survival. A further 3 cases were reviewed and excluded due to lack of EGFR mutation, giving a final study population of 505 patients (30 stage IIIB, and 475 stage IV). Figure 1 Flow chart.

218 cases (43.17%) were identified with survival less than or equal to 24 months, while 287 patients (56.83%) had survival greater than 24 months.

Table 1 summarizes the characteristics of the population included. Average age at diagnosis was 64 years with 60.8% being women and an ECOG 0–1 in 85.72%. With regard to smoking history, only 54% of cases were never smokers, with adenocarcinoma being the most common histology in 95.45%. As to EGFR mutation type, exon 19 deletion was the most common, 55.25%. The second most common was the L858R mutation in exon 21, 28.32%. The third most common was the exon 20 insertion, 4.7%. 22 patients had only de novo EGFR T790M mutation. The T790M was determined as an acquired resistance mutation in 83 patients, 57% of cases with exon 19 deletion and 26% with L858R.

**Table 1:** Baseline characteristics of the global cohort

Characteristics		Total (***N*** = 505)
Age (years)	Mean (standard deviation)	64 (12.2)
Sex	Male	198 (39.2%)
	Female	307 (60.8%)
ECOG *	0	160 (31.8%)
	1	272 (54%)
	2	55 (10.9%)
	3	15 (3%)
	4	2 (0.4%)
Weight loss *	Yes	104 (27.2%)
	No	278 (72.8%)
Smoking history *	Never smoker	269 (54.0%)
	Ex-smoker	168 (33.7%)
	Current smoker	61 (12.3%)
Lives with a smoker *	Yes	43 (24.9%)
	No	130 (75.1%)
Distribution of stages	Stage IIIB	30 (5.9%)
	Stage IV	475 (94.1%)
Histology	Adenocarcinoma	482 (95.5%)
	Other	23 (4.6%)
Type of mutation **	Exon 19 deletion	279 (55.2%)
	L858R	143 (28.3%)
	Exon 20 insertion	24 (4.7%)
	Exon 18 G719X	17 (3.4%)
	S768I	10 (2%)
	Unknown	20 (4%)
	L861Q	2 (0.4%)
	T790M at diagnosis	22 (4.3%)
N° of metastatic sites	0	30 (5.9%)
	1	182 (36.0%)
	2	126 (25%)
	3	85 (16.8%)
	4	48 (9.5%)
	5	23 (4.6%)
	6	2 (0.4%)
	7	3 (0.6%)
	Unknown	6 (1.2%)
N° of patients with metastatic site at diagnosis**	Bone	208 (41.2%)
	Lung	190 (37.6%)
	Pleural effusion	136 (26.9%)
	CNS	85 (16.8%)
	Liver	73 (14.5%)
	Adrenal glands	45 (8.9%)
	Pericardial effusion	11 (2.2%)

* Patients do not add up to the exact total (n 505) due to lack of data

** Patients may add up to more than the total (n 505) since a patient may have one or more mutations or metastatic sites at diagnosis

At the time of diagnosis, the most frequent metastatic locations were bone in 41.2%, lung in 37.6%, and pleural in 26.9% of cases. The most common sites of single metastatic location were bone, 25.3%, lung, 25.3%, and CNS, 14.8%.

### Treatments received

Fifteen patients only received palliative care. The majority of patients who were treated received a TKI in the first line, 76.63% while the other 16.84% received chemotherapy. As a consequence of the inclusion stage IIIB patients, 14 (2.77%) cases received concomitant CT-RT. Four patients received immunotherapy in a clinical trial setting (0.79%).

One hundred and ninety cases (37.62%) did not receive a second line of treatment. In 23.16%, the second line was chemotherapy, while 35.84% received another TKI in second line setting. In second line, the administration of concomitant CT-RT was described in 3 patients, while 14 (2.77%) patients received immunotherapy within a clinical trial. The type of TKI received in each line is summarized in Appendix Table 1. Regarding the number of treatment lines received, 3% of the patients did not receive any treatment, while 34% and 29% received one or two types of therapies, respectively. Less than half of the patients received some line of treatment in a clinical trial setting (40.28%). There were no differences between the survival curves in patients who received first-line treatment with TKIs, chemotherapy, radiotherapy and immunotherapy (log-rank *p*-value = 0.521).

### Overall survival of the whole patient cohort

Median follow-up in our cohort was 42 months (95% CI: 38.5–48.5). Appendix Fig. 1 shows the OS curve for the 505 EGFR mutated patients calculated using the Kaplan-Meier method. Median survival was 27.7 months (95% CI: 24.4–32.8). Using the methodology described by Val Gebski [16], 52% data maturity was observed at 60 months, and 47% at 72 months. Data beyond 72 months was, therefore, considered immature and not interpreted. Applying the criterion of the width of the 95% confidence interval, the results in the Kaplan-Meier curve cannot be considered valid when only 7 subjects remain at risk.

### Results of the univariate analysis

The univariate analysis was performed based on survival less than or equal to 24 months vs. greater than 24 months to relate the different socio-demographic variables, as well as those related to the tumor (Table 2) and the type of treatment received (Appendix Table 2).

**Table 2:** Univariate analysis of survival less than or equal to 24 months vs. greater than 24 months

Characteristics		***N***	≤24 months	> 24 months	OR (95% CI)	p-value
Age (years)	Mean (standard deviation)	505	64.5 (12.7)	64.19 (11.9)	0.99 (0.98–1.01)	0.782
Sex	Male	198	101 (46.3%)	97 (33.8%)	1.69 (1.17–2.42)	0.004
	Female	307	117 (56.7%)	190 (66.2%)		
ECOG *	0–1	432	163 (75.1%)	269 (93.7%)	0.2 (0.11–0.35)	< 0.001
	2–4	72	54 (24.9%)	18 (6.3%)		
Weight loss *	Yes	102	53 (33.3%)	51 (22.9%)	0.59 (0.37–0.93)	0.024
Smoking history *	Never smoker	273	106 (49.3%)	163 (57.6%)	0.80 (0.54–1.19) 0.51 (1.20–1.96)	0.060
	Ex-smoker	168	75 (34.9%)	93 (32.9%)		
	Current smoker	61	34 (15.8%)	27 (9.5%)		
Lives with a smoker *	Yes	43	18 (28.1%)	25 (22.9%)	0.76 (0.37–1.53)	0.446
Distribution of stages	Stage IIIB	30	9 (4.1%)	21 (7.3%)	0.54 (0.24–1.21)	0.133
	Stage IV	475	209 (95.9%)	266 (92.7%)		
Type of mutations**	Exon 19 deletion	279	109 (50.0%)	170 (59.2%)	1.45 (1.01–2.07)	0.039
	L858R	143	59 (27.1%)	84 (29.3%)	1.11 (0.75–1.65)	0.586
	Exon 20 insertion	24	16 (7.3%)	8 (2.8%)	0.36 (0.15–0.86)	0.020
	G719X	17	10 (4.6%)	7 (2.4%)	0.52 (0.19–1.39)	0.141
	S768I	10	5 (2.3%)	5 (1.7%)	0.75 (0.21–2.64)	0.660
	L861Q	2	1 (0.5%)	1 (0.4%)	0.75 (0.05–12.19)	1.000
	Unknown	20	12 (5.5%)	8 (2.8%)	0.49 (0.19–1.22)	0.121
	T790M	146	30 (13.8%)	116 (40.4%)	4.25 (2.70–6.67)	< 0.001
	T790M + Del19	83	12 (5.5%)	71 (24.7%)	5.64 (2.97–10.71)	< 0.001
	T790M + L858R	38	6 (2.7%)	32 (11.1%)	4.43 (1.81–10.80)	< 0.001
N° de metastatic sites	Average (P25; P75)	505	2 (1; 3)	1 (1; 3)	0.75 (0.66–0.86)	< 0.001
N° of patients with metastatic site at diagnosis **	Bone	208	100 (45.9%)	108 (37.6%)	0.71 (0.50–1.01)	0.062
	Lung	190	84 (38.5%)	106 (36.9%)	0.93 (0.65–1.34)	0.713
	Pleural effusion	136	64 (29.4%)	72 (25.1%)	0.80 (0.54–1.19)	0.284
	CNS	85	51 (23.4%)	34 (11.9%)	0.44 (0.27–0.71)	0.001
	Liver	73	44 (20.2%)	29 (10.1%)	0.44 (0.27–0.74)	0.001
	Adrenal glands	45	31 (14.2%)	14 (4.9%)	0.31 (0.16–0.60)	< 0.001
	Pericardial effusion	11	4 (1.8%)	7 (2.4%)	1.33 (0.38–4.62)	0.445

* Patients do not add up to the exact total (n 505) for some variables due to lack of data

** Patients may add up to more than the total (n 505) since a patient may have one or more mutations or metastatic sites

No significant differences were observed in the age at diagnosis between the patients who survived more than 24 months and those who did not, the mean age in both groups being 64 years. There was a higher percentage of women than men with survival more than 24 months (66% vs. 57%, *p* = 0.004). Among patients with long-term survival, 93% had a better PS (ECOG 0–1) compared to 75% for those with survival less or equal 24 months (*p* < 0.001). Weight loss (defined as an unintentional weight loss of more than 5% within a six-month period) was also an important factor for survival, being less frequent in patients who survived more than 24 months than in patients with survived 24 months or less (23 and 33% respectively). No statistical relationship with smoking status and survival was found (p = 0,060) between the two populations. We found a clear relationship with the prognosis according to the type of mutation: the deletion of exon 19 was detected more frequently in cases of long survival (59% vs. 50%, *p* = 0.039), unlike the L858R mutation (29% vs. 27%, *p* = 0.586). Insertion of exon 20 was a negative prognostic factor for survival (7% vs. 3%, *p* = 0.020). The development of the T790M resistance mutation was a favorable factor with both the L858R substitution and the exon 19 deletion (Table 2).

Regarding metastatic sites, the univariate analysis revealed that liver, central nervous system (CNS) and adrenal involvement had the greatest impact on survival. Clear differences were also observed according to the number of metastatic sites, with an average of 2 sites for patients with survival less or equal 24 months vs one site for patients with survival more than 24 months (p < 0.001). Participation in a clinical trial (CT) also occurred more frequently in the population with the longest survival. Among patients with survival over 24 months, 58% received a TKI in first line, 74% in second line, and 82.67% in third line. Increase in survival was significantly related to the number of treatment lines received, with a median of 2 lines [1–4] in those who survived more than 24 months.

### Results of the multivariable analysis

Variables that were significant in the univariate analysis were sex; ECOG; weight loss at diagnosis; presence of exon 19 deletion and exon 20 insertion; appearance of the T790M mutation during treatment; presence of CNS, liver or adrenal metastases; burden of metastatic sites and participation in a clinical trial. In order to prevent selection bias, patients who received no treatment at all (*n* = 15) and those who received osimertinib as first-line treatment (*n* = 5) were excluded from the model.

Variables related to treatments administered after diagnosis, as well as those acquired during follow-up (such as the appearance of the T790M resistance mutation), were not taken into account in the multivariable analysis as the aim of our prognostic model is to understand the probability of survival at time of diagnosis.

After verifying no collinearity (Appendix Table 4) between the independent variables, the maximum model contained the following variables: sex; age; smoking status; TNM staging system; ECOG; weight loss; exon 19 deletion; exon 20 insertion; liver, adrenal and CNS metastases; and total number of metastatic sites. Applying the 1:10 empirical rule [14] when evaluating the number of independent variables to include in the model, up to 21 variables could be entered. An automatic backward regression modeling strategy was performed, eliminating from the maximum model those variables with a significance level *p* > 0.10. In the final model, the variables shown in Table 3 remained, with their corresponding OR (95% CI). For internal validation, 500 re-samplings were carried out by bootstrapping and the number of times each variable is selected are shown (Appendix Table 3).

**Table 3:** Multivariable analysis using the variables included in the final model

Variables in the final model	OR *	p-value	95% CI
**Sex (Female)**	**1.78**	0.017	1.11–2.84
**N° of metastatic sites**	**0.82**	0.033	0.68–0.98
**Adrenal metastases (Yes)**	**0.43**	0.051	0.18–1.00
**CNS metastases (Yes)**	**0.45**	0.016	0.24–0.86
**ECOG (≥2)**	**0.20**	< 0.001	0.10–0.40
**Exon 20 insertion (Yes)**	**0.36**	0.060	0.13–1.05
**Liver metastases (Yes)**	**0.52**	0.063	0.27–1.04
**Constant**	**2.77**	< 0.001	1.64–4.69

* OR: *Odds Ratio; 95% CI: Confidence interval. The maximum model included: sex, age, smoking history, stage, ECOG, weight loss, Exon 19 deletion, Exon 20 insertion, liver metastases, CNS metastases, adrenal metastases, N° of metastatic sites*

**Fig. 2:**
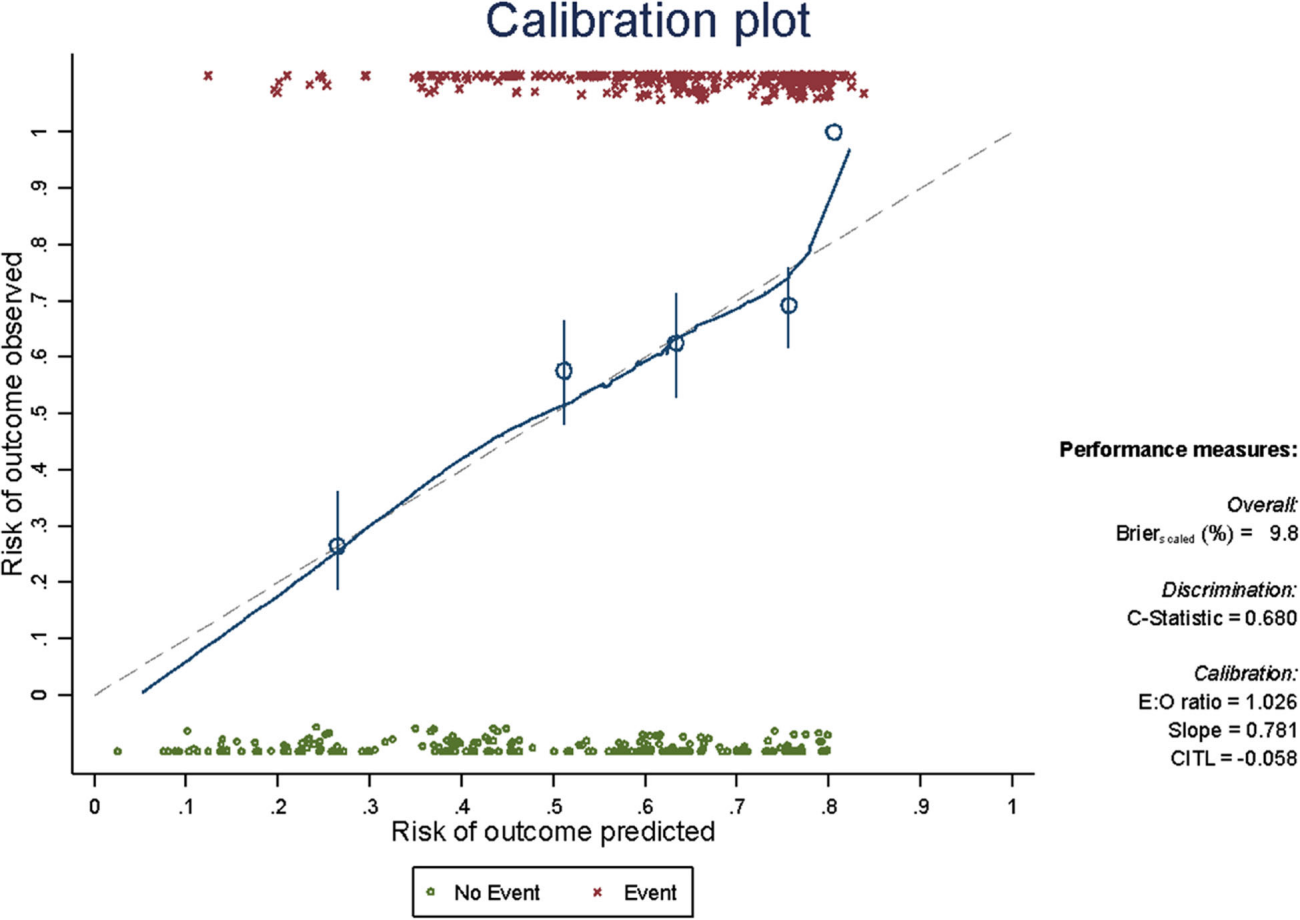
Calibration plot and C-statistic value that measures the discrimination. Perfect calibration is shown on the dotted line, and the fit between expected and observed risks is shown on the solid line. The line fits well in most quintiles (represented by the circles), and only deviates when there are a small number of observations (from a predicted risk of 80%)

**Fig. 3:**
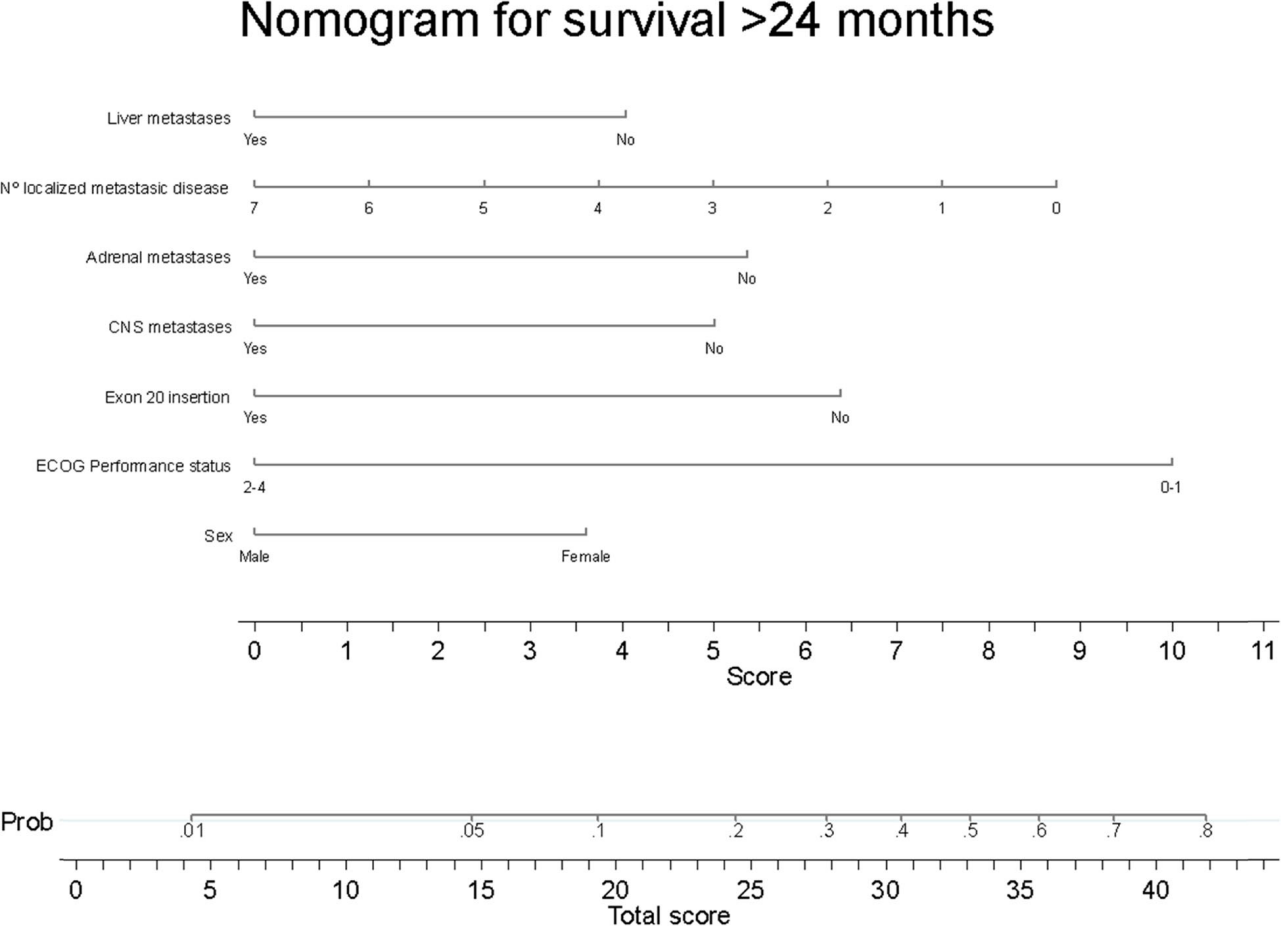
Nomogram of long-term survivors (survival greater than 24 months)

The discrimination ability of the model is good, with a C-index equal to 0.711 (95% CI 0.665–0.757). The C-index of the final model optimism-adjusted for bootstrap is equal to 0.680 (CI 95% 0.627–0.726). The calibration plot (Fig. 2) shows how the calibration line lies almost perfectly along the main diagonal (perfect calibration) except at the ends, which indicates the low number of events and no events at the prediction extremes [13]. Applying the optimism-adjustment for bootstrap, the slope of the calibration curve is equal to 0.781. To facilitate the interpretation and weight of each variable, a nomogram (Fig. 3) was developed. The nomogram provides prognostic information for OS greater than 24 months in a stage IIIB-IV patient with an EGFR mutation. Each of the variables has an associated score, which give a final total score when added together. Drawing a perpendicular line upward from the axis of the overall score gives the model’s predicted probability at the time of diagnosis for this patient to achieve survival greater than 24 months.

## Discussion

After studying the EGFR mutated population of real life patients, we can state that the OS data in our cohort are comparable with other published real world data and have an adequate follow-up [17, 18]. In contrast with other articles reported, our cohort has fewer never smoker patients (54%). Although our data come from daily clinical practice, most patients (85.72%) had a good ECOG 0–1 and a low median number of metastatic sites at diagnosis [1, 2], which may be linked to increasingly early diagnoses with fewer symptoms. Exon 19 deletion is the most frequent mutation in our population, along with a lower frequency of the L858R mutation compared to that published in other series [19]. The most common metastatic site at diagnosis was bone, followed by lung and pleural involvement. The now-standard practice of administering a TKI in first line occurred in our cohort in 76.7% of cases.

Given that clinical studies exclude patients with certain characteristics that could indicate worse prognosis, such as brain involvement or poor ECOG, we believe it is important to evaluate real life data that can have a complementary role in determining the efficacy and safety of treatments. Our study does have several limitations, such as its retrospective nature, with data included by different people and a potential inconsistency or lack of data.

There is also a potential selection bias in terms of inclusion of patients in the registry since only 15 patients did not receive any line of treatment, a percentage that is probably below that of in clinical practice, therefore we excluded this patients from the nomogram. Although the best prognosis has been described in never smokers, in our study, smoking history was not related to survival or prognosis [20]. However, it is true that our population contained fewer never smoker patients. Florescu M. et al. [21] extracted 10 factors related to prognosis from the BR21 study with erlotinib, among them, ECOG, weight loss and smoking. In our study, although data about weight loss was available for few patients, it was a significant factor in the univariate analysis. Participation in a clinical trial (CT) was associated with survival however it is probably subject to confounding factors since these patients have better PS. In line with different data in the literature, the presence of an exon 19 deletion yielded better survival data compared to the L858R mutation in exon 21, as well as the emergence of resistance mutation T790M [22] associated with either of the two sensitive mutations (del19 and L858R). In our univariate analysis, we found a significant association with metastatic spread to the liver, CNS and adrenal glands, conferring worse survival prognosis. Various studies clearly demonstrate worse prognosis with liver involvement and the worst ECOG is related to adrenal involvement [23]. In other studies, bone involvement is related to prognosis in patients with an EGFR mutation who receive an TKI [24]. However, we did not find this last association.

Our study demonstrates that a certain combination of variables at diagnosis can help predict prognosis, since we found six variables associated with a higher probability of survival greater than 24 months. We did not include variables either relating to or appearing during the course of treatment, such as the T790M resistance mutation, since we were looking for a tool that could be used at diagnosis to provide personalized information on survival probability for the patient and their oncologist.

Different publications have found an association between female sex and better prognosis in lung cancer independent of EGFR mutation status. One SLCG publication describes a median OS of 32 months for women and 19 months for men with EGFR mutations, while in non-mutated patients the median OS remained higher in women, 19 vs. 12 months [25]. It is well known that ECOG is related to prognosis. This, along with sex, is the variable most represented in prognostic nomograms, highlighting the importance of its weight. There are few nomograms that predict survival in lung cancer but ECOG is one of the variables in the model developed by Keam B. et al. in EGFR mutated patients to predict progression-free survival (PFS) in those receiving a TKI [24]. The presence of exon 20 insertion [26] confers worse survival compared to common sensitivity mutations (del19 and L858R) without response to currently available TKIs and clear implications for prognosis. In our final model, exon 20 insertion was maintained as a predictive variable of prognosis, unlike exon 19 deletion that loses its level of significance. It is known that a greater number of metastatic sites confers worse prognosis [27]. In one publication on EGFR mutated patients, ECOG ≥2, intra- and extra-thoracic metastases, a greater number of metastatic sites, adrenal and liver metastases, and rapid progression at diagnosis were associated with PFS and OS in the univariate analysis [21]. In the multivariable analysis, only ECOG and rapid tumor progression were still associated with worse PFS. Various studies show the predisposition for brain involvement in EGFR mutated adenocarcinoma [28], this being present at diagnosis in 25% of patients and developing at 3 years in approximately 50% of cases [29]. Given that there are TKIs such as osimertinib that can cross the blood-brain barrier (BBB) [30], this provides a better survival to EGFR mutated patients comparing to wild type.

However, brain involvement is clearly a negative factor that always affects survival [31]. Dissemination to the adrenal glands was maintained as a predictive variable of survival in our model as well as liver involvement which it is well-known its relationship with bad prognosis.

In the literature, it has been described how adrenal involvement confers worse prognosis in both wild type and EGFR mutated lung cancer [27]. A review of 409 patients (not selected for EGFR mutation) found a statistically significant relationship with the presence of intra-abdominal metastases (with particularly poor prognosis due to the presence of adrenal, *p* = 0.011, liver metastases, *p* < 0.001 and intra-abdominal adenopathy, *p* = 0.014) [23]. Similarly, another publication on the TNM staging system described the worst survival with adrenal involvement, independently of EGFR mutation status [32]. It is known that liver involvement is associated with worse prognosis in lung cancer and therefore it is one of the variables present in our nomogram.

We know that the population with the highest probability of survival extracted from the Spanish Registry of Thoracic Tumors of the GECP has the following characteristics: being a woman, absence of exon 20 insertion, absence of brain metastases, absence of liver and adrenal metastases, fewer metastatic locations, and a better functional status 0–1. Given that this group of patients would have a better prognosis at the beginning and a lower hazard of death, it is in this group that we could carry out a study to sequence different TKIs (1st, 2nd and 3rd generation) and chemotherapy. There are various liquid biopsy studies [33] that have observed that, depending on the allelic frequency of the mutation (MAF), it is possible to identify low-risk patients who could be candidates for sequential treatment. In comparison, patients at a higher risk of death would be those who would benefit the most from starting first-line osimertinib therapy.

Therefore, we would be able to combine the variables selected by the nomogram with the molecular determination of the allelic fraction prior to the start of treatment, in order to identify those patients with a lower risk of progression in which it would be possible to sequence the treatments.

## Conclusions

With these six variables -*sex, ECOG, exon 20 insertion, presence of CNS, liver or adrenal metastases and number of metastatic sites-* we have constructed a prognostic nomogram (Fig. 3) with good calibration and discrimination to predict long survival in patients with an EGFR mutation. No other publication exists that only uses patient variables at diagnosis without the therapy initiated in patients with an EGFR mutation. Therefore, this is the first prognostic model that can help predict the probability of long survival before starting therapy.

Given that in our registry almost no patients were able to receive osimertinib in first line (except for 5 patients in a clinical trial) due to its recent approval for that indication, those who survived more than 24 months received first or second generation EGFR TKI and chemotherapy sequences. Therefore, we could select patients for those variables present at diagnosis like being a woman with a good ECOG 0–1, without exon 20 insertion or adrenal, liver or brain metastases and with a low burden of metastatic sites. We believe this population would be the candidate for sequential treatment with different TKIs and chemotherapy to achieve long survival.

Of course, this would need to be tested in randomized clinical trials and it could be interesting to stratify the population based on these variables to randomize treatment sequences with TKIs and chemotherapy vs. osimertinib in first line. It is possible that adding the study of liquid biopsies with quantification of the allelic fraction of the sensitivity mutations and the appearance of the T790M resistance mutation can further improve patient selection to determine the best treatment sequence.

This nomogram could assist clinicians in their daily practice and could be useful to design future clinical trials.

## Supplementary Information

**Additional file 1.**

## Data Availability

The datasets used and analyzed during the current study are available from the corresponding author on reasonable request.

## Abbreviations

CNS: Central Nervous System
CI: Confidence Interval
CT: Clinical Trial
EGFR: Epidermal Growth Factor Receptor
ECOG PS: Eastern Cooperative Oncology Group Performance Status
G: Generation
NSCLC: Non-Small Cell Lung Cancer
OS: Overall Survival
OR: Odds Ratio
PFS: Progression-Free Survival
RCT: Randomized Controlled Trials
SLCG: Spanish Lung Cancer Group
TTR: Thoracic Tumor Registry
TKIs: Tyrosine Kinase Inhibitors
US: United States
