# Supplementary material for: Prognostic model of long-term advanced stage (IIIB-IV) EGFR mutated non-small cell lung cancer (NSCLC) survivors using real-life data
Source: BMC Cancer. 2021 Aug 31;21:977. doi: 10.1186/s12885-021-08713-8 (PMC8406921; doi:10.1186/s12885-021-08713-8)
Supplement: Supplementary file 1 — Additional file 1. [file 12885_2021_8713_MOESM1_ESM.docx]

| Time (months) | Survival function | CI 95% |
| --- | --- | --- |
| 3 | 93.0% | 90.40-94.90 |
| 6 | 86.8% | 83.50-89.40 |
| 9 | 82.9% | 79.29-85.94 |
| 12 | 77.0% | 72.98-80.46 |
| 24 | 55.9% | 51.26-60.33 |
| 36 | 42.4% | 37.55-47.07 |
| 48 | 31.1% | 26.10-36.11 |
| 60 | 23.7% | 18.77-28.98 |
| 72 | 22.0% | 16.98-27.47 |

**
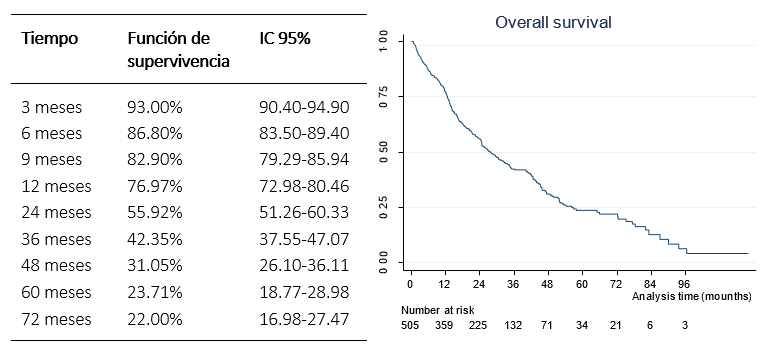
**

**Appendix Fig. 1.** Kaplan-Meier curve for overall survival.

Survival function and 95% CI at different time points (left). Median follow-up was 42 months (IC 95% 38.5-48.5) and median overall survival 27.7 months (IC 95% 24.4-32.8) (right).

**Appendix Table 1**

Distribution of treatments given to patients.

| **Treatments** |  | **Total** |  |
| --- | --- | --- | --- |
| In clinical trial * | Yes | 505 | 199 (40.3%) |
|  | No |  | 295 (59.7%) |
| 1^st^ line of treatment | No treatment | 505 | 15 (3%) |
|  | Chemotherapy |  | 85 (16.8%) |
|  | TKI |  | 387 (76.6%) |
|  | Concomitant CT-RT |  | 14 (2.8%) |
|  | Immunotherapy |  | 4 (0.8%) |
| 2^nd^ line of treatment | No treatment | 505 | 190 (37.6%) |
|  | Chemotherapy |  | 117 (23.2%) |
|  | TKI |  | 181 (35.8%) |
|  | Concomitant CT-RT |  | 3 (0.6%) |
|  | Immunotherapy |  | 14 (2.8%) |
| TKI * | 1^st^ line | 505 | 387 (76.6%) |
|  | 2^nd^ line |  | 181 (35.8%) |
|  | 3^rd^ line |  | 75 (14.9%) |
| TKI received in 1^st^ line | Erlotinib (1^st^ G) | 387 | 103 (27%) |
|  | Gefitinib (1^st^ G) |  | 167 (43%) |
|  | Afatinib (2^nd^ G) |  | 107 (28%) |
|  | Dacomitinib (2^nd^ G) |  | 5 (1%) |
|  | Osimertinib (3^rd^ G) |  | 5 (1%) |
|  | Rociletinib (3^rd^ G) |  | 0 (0%) |
| TKI received in 2^nd^ line | Erlotinib (1^st^ G) | 181 | 55 (30.3%) |
|  | Gefitinib (1^st^ G) |  | 29 (16%) |
|  | Afatinib (2^nd^ G) |  | 22 (12.1%) |
|  | Dacomitinib (2^nd^ G) |  | 1 (0.5%) |
|  | Osimertinib (3^rd^ G) |  | 71 (39.2%) |
|  | Rociletinib (3^rd^ G) |  | 3 (1.7%) |
| TKI received in 3^rd^ line | Erlotinib (1^st^ G) | 75 | 16 (21%) |
|  | Gefitinib (1^st^ G) |  | 14 (19%) |
|  | Afatinib (2^nd^ G) |  | 13 (17%) |
|  | Dacomitinib (2^nd^ G) |  | 0 (0%) |
|  | Osimertinib (3^rd^ G) |  | 28 (37%) |
|  | Rociletinib (3^rd^ G) |  | 4 (5%) |
| Nº of treatment lines | 0 | 505 | 15 (3%) |
|  | 1 |  | 172 (34%) |
|  | 2 |  | 147 (29%) |
|  | 3 |  | 84 (17%) |
|  | 4 |  | 42 (8%) |
|  | 5 |  | 21 (4%) |
|  | 6 |  | 12 (2%) |
|  | 7 |  | 12 (2%) |
|  | Total |  | *Median percentile 50 = 2 P25 1 - P75 3 (min. 0 - max. 7)* |

* Patients do not add up to the exact total (505) due to lack of data.

* G means Generation.

**Appendix Table 2**

Univariate analysis of treatments.

| **Variable** |  | **≤ 24 months** | **> 24 months** | **OR (IC 95%)** | **p** |
| --- | --- | --- | --- | --- | --- |
| TKI in 1^st^ line | Yes | 162 (41.9%) | 225 (58.1%) | 1.25 (0.83-1.90) | 0.283 |
|  | No | 56 (47.5%) | 62 (52.5%) |  |  |
| TKI in 2^nd^ line | Yes | 46 (25.4%) | 135 (74.6%) | 3.46 (2.31-5.19) | < 0.05 |
|  | No | 172 (53.1%) | 152 (46.9%) |  |  |
| TKI 3^rd^ line | Yes | 13 (17.3%) | 62 (82.7%) | 4.34 (2.32-8.13) | < 0.05 |
|  | No | 205 (47.7%) | 225 (52.3%) |  |  |
| Nº of treatment lines | 0 | 13 (86.7%) | 2 (13.3%) |  |  |
|  | 1 | 102 (59.3%) | 70 (40.7%) |  |  |
|  | 2 | 59 (40.1%) | 88 (59.9%) |  |  |
|  | 3 | 34 (40.5%) | 50 (59.5%) |  |  |
|  | 4 | 9 (21.4%) | 33 (78.6%) |  |  |
|  | 5 | 0 (0%) | 21 (100%) |  |  |
|  | 6 | 1 (8.3%) | 11 (91.7%) |  |  |
|  | 7 | 0 (0%) | 12 (100%) |  |  |
|  | Total | p50 1 (1-2) (p25-p75) | p50 2 (1-4) (p25-p75) | 1.86 (1.57-2.20) | < 0.05 |
| Participated in clinical trial * | Yes | 73 (36.7%) | 126 (63.3%) | 1.53 (1.06-2.22) | 0.022 |
|  | No | 139 (47.1%) | 156 (52.9%) |  |  |

* Patients do not add up to the exact total (505) due to lack of data.

**Appendix Table 3**

Number of times each variable is selected through bootstrap resampling.

| **Variables** | **%** |
| --- | --- |
| ECOG | 99% |
| Sex | 83% |
| CNS metastases | 82% |
| Total metastatic sites | 77% |
| Adrenal metastases | 70% |
| Liver metastases | 58% |
| Exon 20 insertion | 49% |
| Exon 19 deletion | 32% |
| Weight loss | 29% |
| Stage | 21% |
| Smoking status | 17% |
| Age | 14% |

**Appendix Table 4**

Collinearity diagnostic.

| **Singular values** | | **Variance decom-position portion** | **Sex** | **Age** | **Smoking history** | **Staging** | **ECOG PS** | **Weight loss** | **Exon 19 deletion** | **Exon 20 insertion** | **Liver metasta-ses** | **CNS metasta-ses** | **Adrenal metasta-ses** | **Nº localized metasta-sic disease** |
| --- | --- | --- | --- | --- | --- | --- | --- | --- | --- | --- | --- | --- | --- | --- |
| 1 | 1.00 | r1 | .007 | .002 | .008 | .002 | .006 | .008 | .008 | .001 | .006 | .006 | .004 | .006 |
| 2 | 2.23 | r2 | .002 | .000 | .000 | .000 | .100 | .007 | .009 | .355 | .038 | .020 | .204 | .001 |
| 3 | 2.42 | r3 | .012 | .001 | .002 | .000 | .016 | .000 | .005 | .372 | .282 | .016 | .108 | .003 |
| 4 | 6.57 | r4 | .349 | .222 | .170 | .117 | .006 | .001 | .114 | .013 | .041 | .008 | .022 | .420 |
| 5 | 10.73 | r5 | .014 | .767 | .006 | .854 | .010 | .002 | .017 | .001 | .001 | .018 | .003 | .150 |
| 6 | 3.92 | r6 | .214 | .002 | .115 | .002 | .030 | .011 | .678 | .137 | .000 | .025 | .000 | .002 |
| 7 | 4.51 | r7 | .235 | .000 | .138 | .022 | .005 | .030 | .081 | .009 | .118 | .000 | .050 | .417 |
| 8 | 2.51 | r8 | .004 | .003 | .010 | .002 | .279 | .000 | .049 | .002 | .003 | .333 | .135 | .000 |
| 9 | 2.63 | r9 | .013 | .000 | .003 | .000 | .233 | .235 | .011 | .003 | .007 | .407 | .022 | .000 |
| 10 | 2.77 | r10 | .005 | .001 | .092 | .000 | .000 | .111 | .020 | .073 | .365 | .007 | .322 | .000 |
| 11 | 2.90 | r11 | .145 | .001 | .442 | .000 | .010 | .017 | .007 | .004 | .025 | .051 | .077 | .000 |
| 12 | 3.01 | r12 | .000 | .001 | .014 | .000 | .304 | .578 | .002 | .030 | .115 | .109 | .052 | .000 |

* Condition number = 10.73
